# The Modulation of Chitosan-DNA Interaction by Concentration and pH in Solution

**DOI:** 10.3390/polym11040646

**Published:** 2019-04-09

**Authors:** Fangqin Ma, Yanwei Wang, Guangcan Yang

**Affiliations:** Department of Physics, Wenzhou University, Wenzhou 325035, China; ma1731326253@163.com (F.M.); wangyw@wzu.edu.cn (Y.W.)

**Keywords:** chitosan, DNA compaction, pH regulation, gene delivery

## Abstract

Chitosan has been widely used to prepare a DNA carrier for highly efficient and non-toxic gene therapy. In the present study, we investigated DNA charge neutralization and compaction by chitosan in solutions of various pH levels by dynamic light scattering (DLS), magnetic tweezers (MT), and atomic force microscopy (AFM). We found that when chitosan concentration is higher than a critical value (0.2 µM), corresponding the ratio of phosphate and NH_2_ in chitosan k=1.9, the electrophoretic mobility of DNA-chitosan complex maintains an almost constant value when pH of solution is less 6.5, the isoelectric point of chitosan. Then it decreases with increasing pH of solution. However, when chitosan concentration is lower than the critical value, the mobility of the complex increases with pH in the range of acidity and reaches the maximum when the pH of the solution approaches the isoelectric point of chitosan. It finally decreases with increasing pH in solutions. The corresponding condensing force of the DNA-chitosan complex measured by single molecular MT changes accordingly with its charge neutralization in the same solution concentration (20 µM) and is consistent with the DLS measurements. This phenomenon might be related to the weakening interaction between DNA and chitosan in low pH solutions, and is verified by measuring the ratio of free chitosan to DNA complex in solutions. We also observed the various morphologies of DNA-chitosan complexes, such as ring, rod, flower, braid, and other structures, under different degrees of deacetylation, molecular weight, solution concentration and pH in solutions by AFM.

## 1. Introduction

DNA is a negatively-charged long-chain organic polymer. In a living cellular system, DNA molecules are confined into a small space that contains proteins and various small ions to compensate the negative charge of DNA to overcome Coulombic repulsion. The long-chain molecules are highly packed in various organisms from viruses to eukaryotic cells in order to store, transport and preserve the genetic material. For example, DNA is compacted into chromatin by combining with positively-charged histones in the nuclear matrix of nucleus in eukaryotic cells [1]. Thus, understanding DNA compaction involving like-charge attraction is important for fundamental biological processes such as chromosome assembly. On the other hand, introducing foreign DNA into cells is also widely used in biotechnology and has received considerable attention in medicine for gene therapy [2,3,4,5,6]. In that case, we need an effective transfection vector that is able to compact DNA, protect it against degradation, and deliver DNA into cells specifically and efficiently. The first step in the process is the complexation between DNA and the oppositely charged agents, including many polyvalent cations, proteins, and positively-charged polyelectrolytes [7,8,9,10,11,12,13,14,15,16,17].

Chitosan is an alkaline polysaccharides with positively charged cationic groups, and is easily biodegradable and highly biocompatible [18]. It has been widely used for a polymeric controlled drug delivery system, and is a promising non-viral DNA vehicle for the development of highly efficient, non-toxic gene therapy [19,20,21,22,23,24]. Chitosan is obtained by deacetylation of chitin and the degree of N-deacetylated determines the charge amount of chitosan [25]. In addition, the molecular weight or the chain length of chitosan is another parameter affecting the DNA complexation ability of chitosan [26]. In general, the larger the molecular weight of chitosan is (implying higher viscosity), the greater the hydrodynamic radius of its DNA-complex is, which corresponds to deficiency for delivery of drug to target cells. However, if the chitosan-DNA complex is compacted too tightly, it is less favorable for DNA release [21,27,28]. We have to balance all the factors including molecular weight, degree of polymerization, and degree of deacetylation to form an appropriate structure and size of chitosan-DNA complex as a gene delivery vehicle [26].

The positively-charged cationic groups of chitosan can be modulated by adjusting the pH of solution, a very promising feature for gene delivery. The pKa value of the amino group of chitosan is about 6.5, implying that it is a polycation electrolyte in acidic and neutral solutions and almost neutral in basic solutions [29,30,31]. In other words, the pH of solution affects the degree of deacetylation of chitosan, thereby is related to the total charge of chitosan. It has been shown that the pH of chitosan solution affects the stability, hydrodynamic radius, and complexation mass concentration ratio of DNA-chitosan complex significantly [32,33,34,35]. In spite of the encouraging advancement, the physiochemical properties of chitosan/DNA complexes have not understood extensively, especially at the single molecular level. In the present study, we investigated the electrostatic, mechanical features, and morphology of chitosan-DNA complexes by dynamic light scattering (DLS), single molecular magnetic tweezers (MT), and atomic force microscopy (AFM). We found that the electrophoretic mobility of the DNA-chitosan complex first increases and then decreases with the increase of pH of solution under the condition of low chitosan concentration. The condensing force of DNA measured in the magnetic tweezers experiment was consistent with the observation under the same conditions. It is expected that our work is helpful in understanding the interaction between chitosan and DNA underlying gene transfection.

## 2. Materials and Methods

### 2.1. Materials

Double-strand λ-phage DNA (48502 bp) was purchased from New England Biolabs (New England Biolabs, Ipswich, MA, USA), and its initial concentration was 500 ng μL^−1^. The final DNA concentration in AFM and DLS is 1 ng μL^−1^. Tris buffers (1 mM) of different pH was used as both stock solution and experimental solution in MT, DLS, and AFM. Chitosan oligosaccharide lactate (average *Mn* = 5000, MW:340.30 g/mol) was purchased from Sigma-Aldrich (Sigma-Aldrich, St. Louis, MO, USA) and was used without further purification. Purified water was obtained from a Milli-Q system (Millipore, Billerica, MA, USA). Antidigoxigenin was purchased from Roche Diagnostics (Sigma-Aldrich, Darmstadt, Germany).

### 2.2. Dynamic Light Scattering

The electrophoresis-mobility measurements were carried out by using a DLS device of Malvern Zetasizer Nano ZS90 (Malvern Instruments Ltd, Worcestershire, UK) equipped with the patented M3-PALS technique. In the measurement of DLS and EM, we adjusted pH of 1 mM tris buffer to a specific value by adding a small amount of highly concentrated HCl or NaOH solution. We used the buffer to dissolve solid state chitosan to obtain 200 µM chitosan solution. Chitosan solution with a specific concentration in our experiment can be obtained by adding more Tris buffer with the same pH to the solution. Then we added 2 µL DNA of 500 ng μL^−1^ to the chitosan solution (1 mL) at different pH values. The final DNA concentration in the solution is 1 ng∙μL^−1^. All measurements were achieved after 30 min incubation at room temperature. The sample cell was kept at 25 °C of temperature.

### 2.3. Atomic Force Microscopy

The sample preparation process for atomic force microscopy is briefly described as follows: Mica was cut into about 1 cm^2^ square pieces and attached to glass slides. They were used as substrates for DNA adsorption, and their surfaces were always freshly cleaved before use. For each sample, a drop of mixture solution of chitosan-DNA about 50 µL was deposited on a freshly cleaved mica surface for 5 min. Then, the surface was rinsed with distilled water and dried with a gentle flow of nitrogen gas. All chemical agents were used as received and all measurements were repeated at least three times to obtain consistent results. All the prepared samples were scanned by AFM (JPK Instruments AG, Berlin, Germany) in AC mode. A silicon AFM probe (NCHR-50, NanoWorld Corporation, Neuchâtel, Switzerland) with aluminum coating was used and its spring coefficient and the resonance frequency are 42 N/m and 320 kHz, respectively. The imaging area is 5 µm × 5 µm and the scan rate is 1.0 Hz. Each image obtained has solution of 512×512 pixels (4–6 nm/pixel).

### 2.4. Tethering DNA by Magnetic Tweezers

The DNA molecule for tethering by transverse magnetic tweezers must be bound at one end to an immobile support (the glass sidewalls) and at the other end to a paramagnetic bead [36]. Thus, one end of DNA is modified by digoxigenin so as to attach the anti-digoxigenin-coated glass sidewall; the other end is modified with biotin to bind avidin-coated magnetic beads. Specifically, about 0.5 µL stock solution of magnetic beads coated with streptavidin (M-280, Dynal Biotech, Oslo, Norway) was gently mixed with 0.5 µL modified DNA for 30 min to form DNA–bead constructs in 200 µL buffer solution.

**Figure 1:**
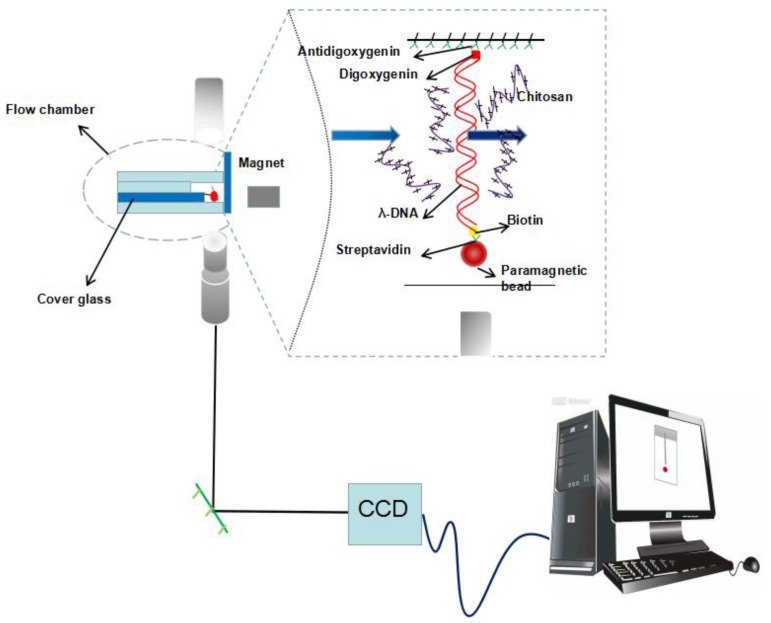
A schematic diagram of magnetic tweezers.

A transverse MT system (Figure 1) is set up on an inverted microscope (Nikon TE2000U, Japan) monitoring the dynamic process of tethering DNA. The detail of setup can be found in our previous work [11]. The coverslips were polished, washed, dried, and then the polished sides were treated with a sigmacote solution. A cover glass slide is glued on a glass slide to serve as the sidewall to anchor an end of DNA. As shown in the left of Figure 1, the flow cell is divided into four layers, and the middle two layers are slightly indented to the top and bottom layers. A syringe pump is used to control the inflow and outflow of the sample cell. The cell with a polished sidewall was dealt with anti-digoxygenin at first and then was rinsed with PBS containing 5 mg ml-1 BSA at pH 8.0. DNA-bead constructs were flushed into the cell, then a side wall–DNA-paramagnetic bead structure was formed, as shown in Figure 1. After the attachment of DNA-bead to the sidewall, the PBS buffer was eluted by injecting Tris buffer. The same Tris buffer was used in the AFM and DLS experiment. A permanent magnet controlled by a micromanipulator system (MP-285, Sutter Instruments (Novato, CA, USA) is used to exert a force on the paramagnetic bead to pull tethered DNA. The movement of paramagnetic bead was recorded by a CCD camera in real-time. 

The chitosan solution with specified pH and concentration for DNA tethering was prepared by the same protocol described in DLS measurement. We introduce the chitosan solution into the flow cell by a syringe pump. In a typical measurement, we pull DNA to its maximal length (about 16 µm) by moving the magnet close to the paramagnetic bead so as to execute a force of more than 10 pN. Then, we use MP-285 to move the magnet slowly away to lower the force to a needed value to observe the conformational change of DNA.

## 3. Results and Discussions

### 3.1. Electrophoretic Mobility and Particle Size of DNA-Chitosan Complex

**Figure 2:**
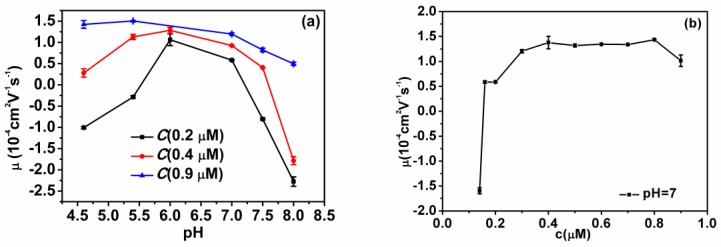
(**a**) Electrophoretic mobility of DNA-chitosan complexes as a function of pH of solutions at various chitosan concentrations (0.2 µM, 0.4 µM, 0.9 µM; corresponding, k are 1.9, 3.8 and 8.55). (**b**) Electrophoretic mobility of DNA-chitosan complexes as a function of chitosan concentration at pH = 7. The concentration of DNA is 1 ng µL^−1^.

The measured electrokinetic properties of DNA-chitosan complexes at various pH and chitosan concentrations in solutions are shown in Figure 2. In Figure 2a, the electrophoretic mobility (EM) of the complex is plotted versus pH of solution at different chitosan concentrations. With the increasing pH in solutions, we can see the EM of the chitosan-DNA complex changes slowly at high chitosan concentration. For example, the EM decreases gradually from 1.46 × 10^−4^ cm^2^v^−1^s^−1^ at pH = 4.6 to 0.7 × 10^−4^ cm^2^v^−1^s^−1^ at pH = 8 when the concentration of chitosan is fixed to 0.9 µM. On other hand, EM changes in a wider range at low chitosan concentration when we adjust pH of solution. In the case of 0.2 µM chitosan concentration, the EM is −1.001 × 10^−4^ cm^2^v^−1^s^−1^ at pH = 4.6, then climbs up to 0.582 × 10^−4^ cm^2^v^−1^s^−1^ at pH = 7, and then goes down to −2.275 × 10^−4^ cm^2^v^−1^s^−1^ when pH goes to pH = 8. The range of variation is about 3.0, much larger than the case of high chitosan concentration. In addition, we observed a new tendency. When the chitosan concentration becomes lower, the EM of the chitosan-DNA mixtures reaches a maximum near the isoelectric point of chitosan. The smaller the concentration is, the clearer this tendency appears. In Figure 2b, the EM of DNA-chitosan complex is plotted against the concentration of chitosan when pH is fixed at 7, slightly above the isoelectric point of chitosan. We can see that the EM increases with the concentration of the solution. It also changes from a negative value to a positive value. The EM of free DNA is negative since the ionization of phosphates of backbone of DNA chain in solution. When chitosan is added, the phosphates attract the positively charged chitosan in solution to neutralize their charges. The extent of neutralization is related with the concentration of chitosan and pH of solution. When pH of solution is in the range of acidity and chitosan concentration of chitosan is low, the charge of DNA is dominant and the EM of DNA–chitosan complexes is negative. In high concentration of DNA, chitosan can overcompensate the charge of DNA leading to the occurrence of charge inversion, implying EM of DNA-chitosan complex becomes positive rather than negative, a charge reversal of DNA. In this case, EM stays at an almost fixed value of around 1.3 × 10^−4^ cm^2^v^−1^s^−1^ when chitosan concentration varies in the range from 0.3 to 0.8 µM, corresponding k from 2.85 to 7.6 (here k is defied; it is the ratio of phosphate and NH_2_ in chitosan. Moreover, there are two positively-charged amino groups in the monomer of chitosan). However, when we increase concentration of chitosan further to 0.9 µM (k=8.55), the EM drops rapidly to 1.0×10^−4^ cm^2^v^−1^s^−1^, implying the reentrant due to Coulomb repulsion. 

**Figure 3:**
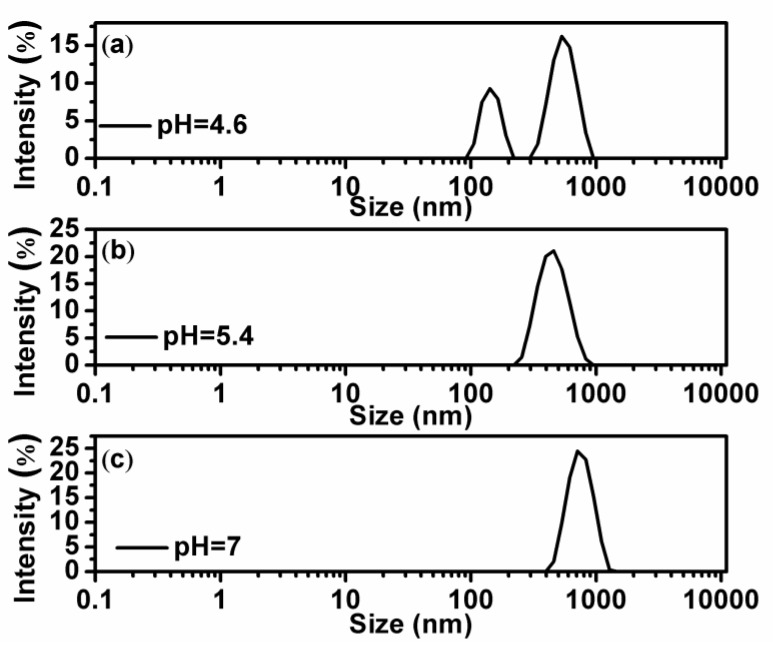
(**a**–**c**) are the size distribution of the DNA-chitosan complex at pH = 4.6, pH = 5.4, and pH = 7, respectively, with a fixed chitosan concentration of 0.2 µM (k=1.9).

**Figure 4:**
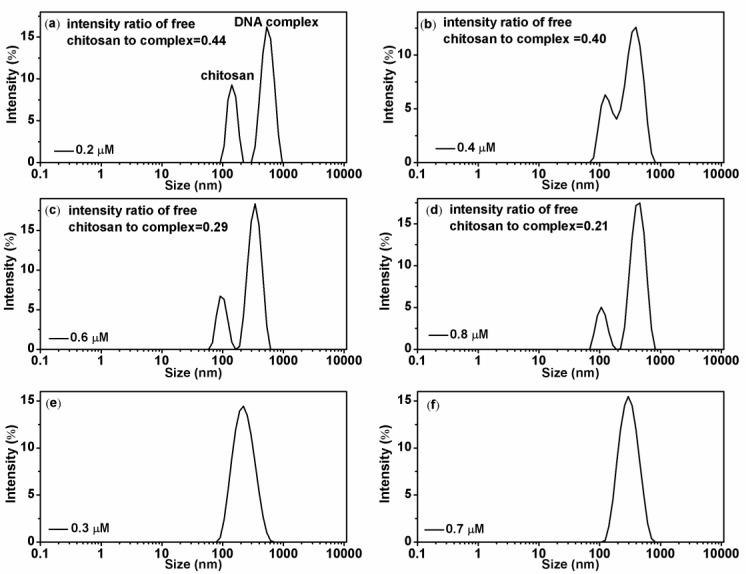
(**a**–**d**): Chitosan-DNA complexes size distribution with different chitosan concentrations (0.2 µM, 0.4 µM, 0.6 µM and 0.8 µM; corresponding, k are 1.9, 3.8, 5.7 and 7.6) at pH = 4.6, intensity ratio of free chitosan to complex are 0.44, 0.40, 0.29, and 0.21, respectively. (**e**,**f**): size distributions at pH = 6, the concentration of chitosan is 0.3 µM and 0.7 µM (k are 2.85 and 6.65), respectively.

In order to explore the mechanism of anomaly at low chitosan concentration, we measured the particle sizes in solutions by dynamic light scattering. The results are shown in Figure 3 and Figure 4. Figure 3 shows the particle size variation of DNA and chitosan with increasing pH of solution under the condition of low chitosan concentration (0.2 µM). When pH = 4.6, as shown in Figure 3a, we can see two discrete peaks corresponding to free chitosan and DNA-chitosan complex, respectively. Obviously, a peak centered at about 150 nm represents free chitosan and the dominant peak centered at about 600 nm corresponds to DNA-chitosan complex. The distribution of free DNA particle size at pH = 4.6 is centered at 550 nm and its width is about 200 nm, which overlapped with the size of DNA-chitosan complex. Thus, we are unable to distinguish free DNA from DNA-chitosan complex in solution by the measurement of particle size. In this case, a large fraction of chitosan is free in solution and is able to be detected by dynamic light scattering, implying that the interaction between DNA and chitosan is quite weak. When we increase pH of solution to 5.4, as shown in Figure 3b, two peaks belonging to chitosan and complex merge in one peak centered at about 500 nm. The observation means that the free chitosan still exists in solution but its fraction in solutes is very small, only shifts the center of dominant complex-peak in a negative direction slightly. If pH of solution goes up further, we can only see that dominant complex-peak centered at about 600 nm but no trace of free chitosan, as shown in Figure 3c. Thus, we conclude that the interaction between DNA and chitosan is dependent on the pH in solution, and acidic environment has a negative effect on the binding of chitosan to DNA. On the other hand, concentration of chitosan is also important even in an acidic environment.

Figure 4 shows the effect of chitosan concentration to its binding to DNA. Figure 4a–d show the particle sizes in solution with increasing chitosan concentration at a fixed pH = 4.6. We can see that more and more free chitosan molecules bind to DNA with increasing concentrations. More specifically, the intensity ratio of free chitosan to DNA-chitosan complex decreases monotonically from 0.44 for 0.2 µM chitosan concentration to 0.21 for 0.8 µM chitosan concentration. It means that the binding of chitosan to DNA exhibit some kind of cooperation effect, in other words, the existing binding of chitosan to DNA recruits more chitosan molecules to the vicinity of complex. The phenomenon appears much notably under the condition of low pH. When we increase the pH of solution, the recruitment effect weakens and finally disappears, as shown in Figure 4e,f, in which at pH = 6, the peak of free chitosan is unable to be identified.

### 3.2. Condensing Force of Tethered DNA Induced by Chitosan

To explore the interaction mechanism between chitosan and DNA, we measured condensing forces of DNA-chitosan complexes by magnetic tweezers at various acidities and chitosan concentrations. DNA compaction is the process of one DNA chain going from free extensible state to a more compactly ordered structure. The DNA compaction involves like-charge attraction and related charge compensation or neutralization. In the previous section, we measured the electrophoretic mobility of DNA in solutions and found the modulation of pH on the interaction of chitosan and DNA. Now we try to find out whether the same effect is valid on DNA compaction by pulling DNA- chitosan complexes in a flow cell with a home-made magnetic tweezers. In the setup, we can see the tethered DNA compaction and measure the tethering force simultaneously when flushing chitosan solution into the flow cell. 

**Figure 5:**
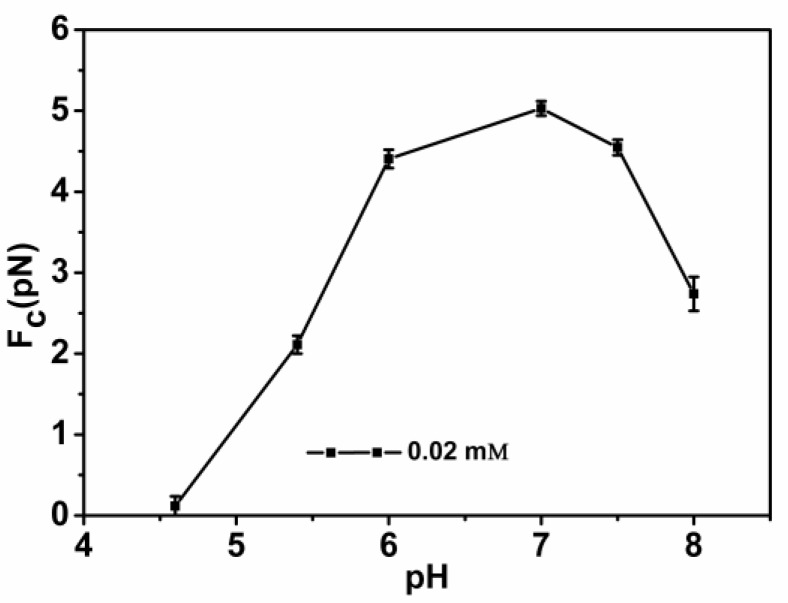
DNA condensing force as a function of the different pHs of solutions with a fixed chitosan concentration (20 µM).

**Figure 6:**
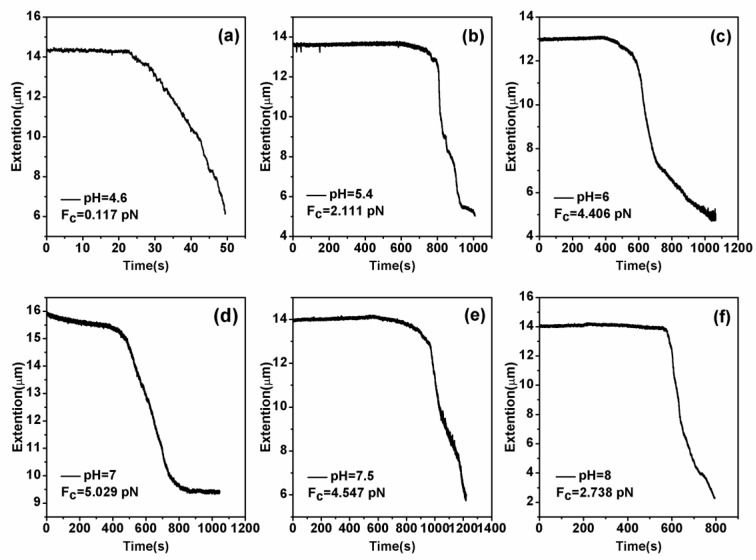
DNA extension as a function of pulling time. (**a**–**f**): The cases of different pHs (pH = 4.6, pH = 5.4, pH = 6, pH = 7, pH = 7.5 and pH = 8) with a fixed chitosan concentration 20 µM.

**Figure 7:**
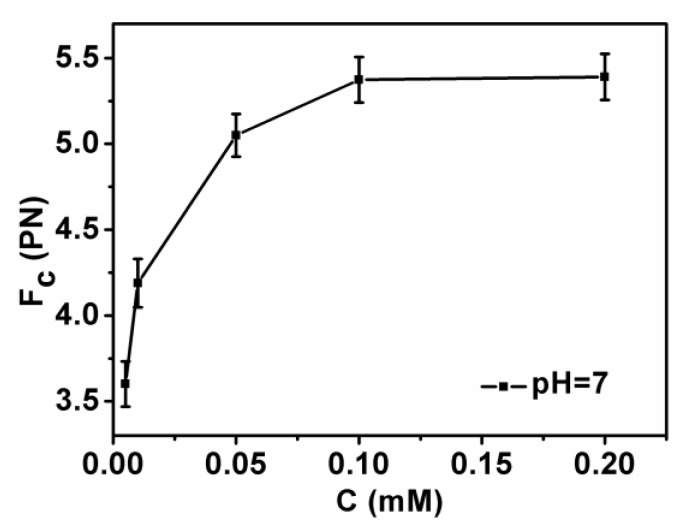
DNA condensing force as a function of chitosan concentration with a fixed pH = 7.

DNA compaction causes a continuous shrinking of extension when magnetic force (*F*) decreases below the condensing force, *Fc*. The results are summarized in Figure 5. In Figure 5, when the concentration of chitosan is constant, the tension of the DNA varies with the pH of the solution. We can see that as the pH increases, the tension of the DNA first increases and then decreases. It can be clearly seen that at pH = 7, the maximum tension of DNA is *F* = 5.029 pN, indicating that the cohesion at this time is the largest. It is consistent with the trend of the curve in the upper in Figure 2. The details of DNA shrinking are shown in Figure 6a–f by pulling the magnet beads at various pH solutions. It can be seen from the figure that when the concentration of chitosan is 20 µM, the reaction is very fast at different pHs. If we start timing when the solution is passed into the sample cell until we stop recording time, it can be seen that the two images are completely different. Figure 7 shows the change of condensing force with chitosan concentration when we fixed the pH of solution. It is remarkable that the force increases with the increase of chitosan concentration, and finally tends to be saturated. 

### 3.3. DNA-Chitosan Morphology by Atomic Force Microscopy

**Figure 8:**
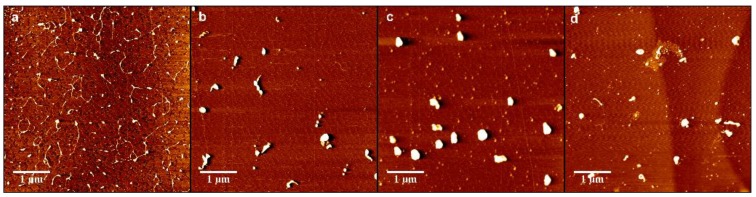

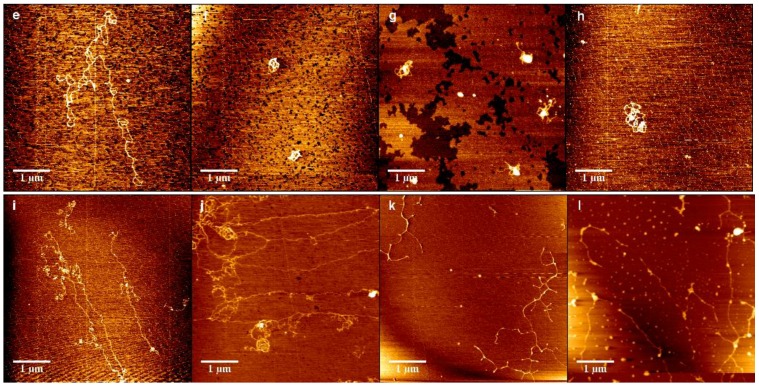
Atomic force images of DNA-chitosan complexes at different chitosan concentrations and pHs. The concentration of Figure (**a**–**d**) chitosan is 0.1 µM, 0.15 µM, 0.35 µM, 0.45 µM, (k=0.95,1.43,3.33,4.28) respectively, pH = 4.6. Similarly, in (**e**–**h**), the concentration of chitosan is 0.15 µM, 0.2 µM, 0.25 µM, 0.3 µM, (k=1.43,1.9,2.38,2.85), respectively, pH = 7. In (**i**–**l**), the concentration of chitosan is 0.2 µM, 0.26 µM, 0.28 µM, 0.36 µM, (k=1.9,2.47,2.66,3.42), respectively, pH = 8.

In order to further analyze the interaction between DNA and chitosan, we imaged the change in the morphology of DNA-chitosan complex by means of AFM. Figure 8a–d show the morphology of DNA-chitosan complex pH = 4.6 with increasing chitosan concentrations, from 0.1 µM, 0.15 µM, 0.35 µM, to 0.45 µM. In Figure 8a we can see DNA condensation by chitosan loosely since some extensible DNA with some knots can be found in the image. When the concentration of chitosan is increased slightly to 0.15 µM, as shown in Figure 8b, DNA-complexes rapidly becomes compact globules with dimensions about 140 nm. When the concentration of chitosan grows further up to 0.35 µM, spherical globules are still the common compact structures, as in Figure 8c, while the size of globules increases to about 300 nm. In the case of high concentration of 0.45 µM, however, as shown in Figure 8d, the spherical globules become various compact structures, such as rods, rings, flowers, and braids in addition of globules, corresponding to possible not so compact DNA particles. This observation by AFM is consistent to the measurement of electrophoretic mobility of DNA-chitosan complex, presented in Figure 2, in which the EM at pH = 4.6 increases monotonously and crosses zero point, resulting in DNA compaction becomes tighter initially and then loose slightly due to Coulombic repulsion. The complete charge neutralization of DNA corresponds to the maximum of compactness of DNA-chitosan complexes as we can expect. The morphology variation of DNA-chitosan complex at pH = 7 demonstrates a similar process even more obviously, as shown in Figure 8e–h. In Figure 8e, we can see that the DNA molecules are well separated and show relaxed coils on the surface at 0.15 µM chitosan concentration. In Figure 8f,g, DNA becomes more compact with some threads still visible, implying the DNA condensing capability of chitosan is weaken significantly at a pH higher than its isoelectric point. DNA morphology becomes even looser when chitosan concentration goes up further, as shown in Figure 8h. When pH reaches 8, DNA is hard to be condensed by chitosan, as presented in Figure 8i–l. In Figure 8i,j, DNA is well separated and loosely distributed on mica surfaces, corresponding to chitosan concentration 0.2 and 0.26 µM. DNA is slightly condensed by chitosan at its concentration 0.28 µM, shown in Figure 8k. In Figure 8l the compactness of DNA is almost unvaried when chitosan concentration goes up to 0.36 µM.

## 4. Conclusions

Through atomic force microscopy, dynamic light scattering and magnetic tweezers, we have come to the conclusion that chitosan is sensitive to the pH of solutions, and its charge is easily modulated by acidity, affecting the compaction, morphology and charge of chitosan-DNA complex. As the pH of solution is lower, the amino group on the chitosan backbone undergoes protonation under acidic conditions, and the charge on chitosan chain is redistributed, increasing the charge density of the chitosan. When chitosan concentration is high enough (>0.9 µM), the electrophoretic mobility of the DNA-chitosan complex fluctuates in a very small range under low pH conditions, then goes down gradually with increasing pH of solution. This is expected since the positive charge of chitosan decreases when pH of solution goes cross its isoelectric point. However, the electrophoretic mobility of DNA-chitosan complexes increases with increasing pH in the range of acidity when concentration of chitosan is lower than the critical value. After passing its maximum located about the isoelectric point of chitosan, and it finally decreases with increasing pH in solutions, similar to the previous case. This finding is also confirmed by measuring the corresponding condensing force of the DNA-chitosan complex, which varies in the same way as the mobility. We attribute the observation to the weakening of interaction between DNA and chitosan in low pH solutions. This inference is consistent to the measurement of the ratio of free chitosan to DNA complex in solutions.
